# Predominant cerebral cytokine release syndrome in CD19-directed chimeric antigen receptor-modified T cell therapy

**DOI:** 10.1186/s13045-016-0299-5

**Published:** 2016-08-15

**Authors:** Yongxian Hu, Jie Sun, Zhao Wu, Jian Yu, Qu Cui, Chengfei Pu, Bin Liang, Yi Luo, Jimin Shi, Aiyun Jin, Lei Xiao, He Huang

**Affiliations:** 1 Bone Marrow Transplantation Center, The First Affiliated Hospital, School of Medicine, Zhejiang University, Hangzhou, China; 2 Shanghai SiDanSai Biotechnology Limited Company, Shanghai, China; 3 Department of Hematology, Beijing Tiantan Hospital, Capital Medical University, Beijing, China; 4 Department of Hematology, the First Affiliated Hospital, Wenzhou Medical University, Wenzhou, China

**Keywords:** Chimeric antigen receptor-modified T cells, CD19, Acute lymphocytic leukemia, Cytokine release syndrome, Blood-brain barrier

## Abstract

Chimeric antigen receptor-modified (CAR) T cells targeting CD19 (CART19) have shown therapeutical activities in CD19+ malignancies. However, the etiological nature of neurologic complications remains a conundrum. In our study, the evidence of blood-brain barrier (BBB)-penetrating CAR T cells as a culprit was revealed. A patient with acute lymphocytic leukemia developed sustained pyrexia with tremors about 6 h after CART19 infusion, followed by a grade 2 cytokine release syndrome (CRS) and neurological symptoms in the next 3 days. Contrast-enhanced magnetic resonance showed signs of intracranial edema. Lumbar puncture on day 5 showed an over 400-mmH_2_O cerebrospinal pressure. The cerebrospinal fluid (CSF) contained 20 WBCs/μL with predominant CD3+ T cells. qPCR analysis for CAR constructs showed 3,032,265 copies/μg DNA in CSF and 988,747 copies/μg DNA in blood. Cytokine levels including IFN-γ and IL-6 in CSF were extremely higher than those in the serum. Methyprednisone was administrated and the symptoms relieved gradually. The predominance of CART19 in CSF and the huge discrepancies in cytokine distributions indicated the development of a cerebral CRS, presumably featured as CSF cytokines largely in situ produced by BBB-penetrating CAR T cells. For the first time, we reported the development of cerebral CRS triggered by BBB-penetrating CAR T cells.

**Trial registration: **ChiCTR-OCC-15007008.

**Electronic supplementary material:**

The online version of this article (doi:10.1186/s13045-016-0299-5) contains supplementary material, which is available to authorized users.

Chimeric antigen receptor-modified (CAR) T cells targeting CD19 (CART19) have shown therapeutical activities in refractory/relapsed acute lymphocytic leukemia (ALL) [1–4]. Neurologic complications were reported in several trials [5–8]; however, the etiological nature still remains a conundrum. In our recent CART19 clinical trial (ChiCTR-OCC-15007008), the evidence of blood-brain barrier (BBB)-penetrating CAR T cells as a culprit was revealed.

An enrolled female patient of BCR/ABL p210(+) refractory/relapsed ALL with previous recurrence of central nervous system lymphoma (CNSL) was infused with autologous CART19 after conditioning chemotherapy with fludarabine and cyclophosphamide (detailed patient and methodological information was included in Fig. 1a and Additional file 1). CART19 preparation and infusion were described in Fig. 1b, c and Additional file 2 [8]. About 6 h after CART19 infusion, the patient developed sustained pyrexia with tremors (Fig. 2a). Three days later, the patient complained of headache, vomit, and recurrent right-sided facial and limb paresis with a homolateral blurred vision and defective visual field. Physical examination showed decreased myodynamia (grade 3), high blood pressure, and positive Babinski and Kernig signs. Papilloedema was observed by ophthalmoscope. Contrast-enhanced magnetic resonance imaging showed signs of intracranial edema (Fig. 2b). Serum cytokines including INF-γ, IL-6, and IL-10 elevated synchronously, supporting a grade 2 cytokine release syndrome (CRS) (Fig. 2c). The cerebral symptoms were not relieved by a bolus infusion of mannitol. Lumbar puncture on day 5 showed an over 400-mmH^2^O cerebrospinal pressure. The cerebrospinal fluid (CSF) contained 20 WBCs/μL and 4.0 g/μL protein (Fig. 2d). CD3+ T cells were predominant in CSF, with few CD19+ B cells (Fig. 2e) which excluded CNSL. qPCR analysis for CAR construct showed 3,032,265 copies/μg DNA in CSF and 988,747 copies/μg DNA in blood. Cytokine levels in CSF were extremely higher than those in the serum, with IFN-γ at 2977 versus 152 pg/ml and IL-6 at 8475 versus 46 pg/ml (Fig. 2c). Methyprednisone was administrated at 3 mg/kg/day since day 5, and the symptoms relieved gradually. By day 9, all cerebral symptoms and signs disappeared, and serum IFN-γ and IL-6 levels decreased to normal ranges. Then, methyprednisone was de-escalated and tapered on day 14. The patient achieved complete remission (CR) with minimal residual disease (MRD) negative 10 days after CART19 infusion.

**Fig. 1:**
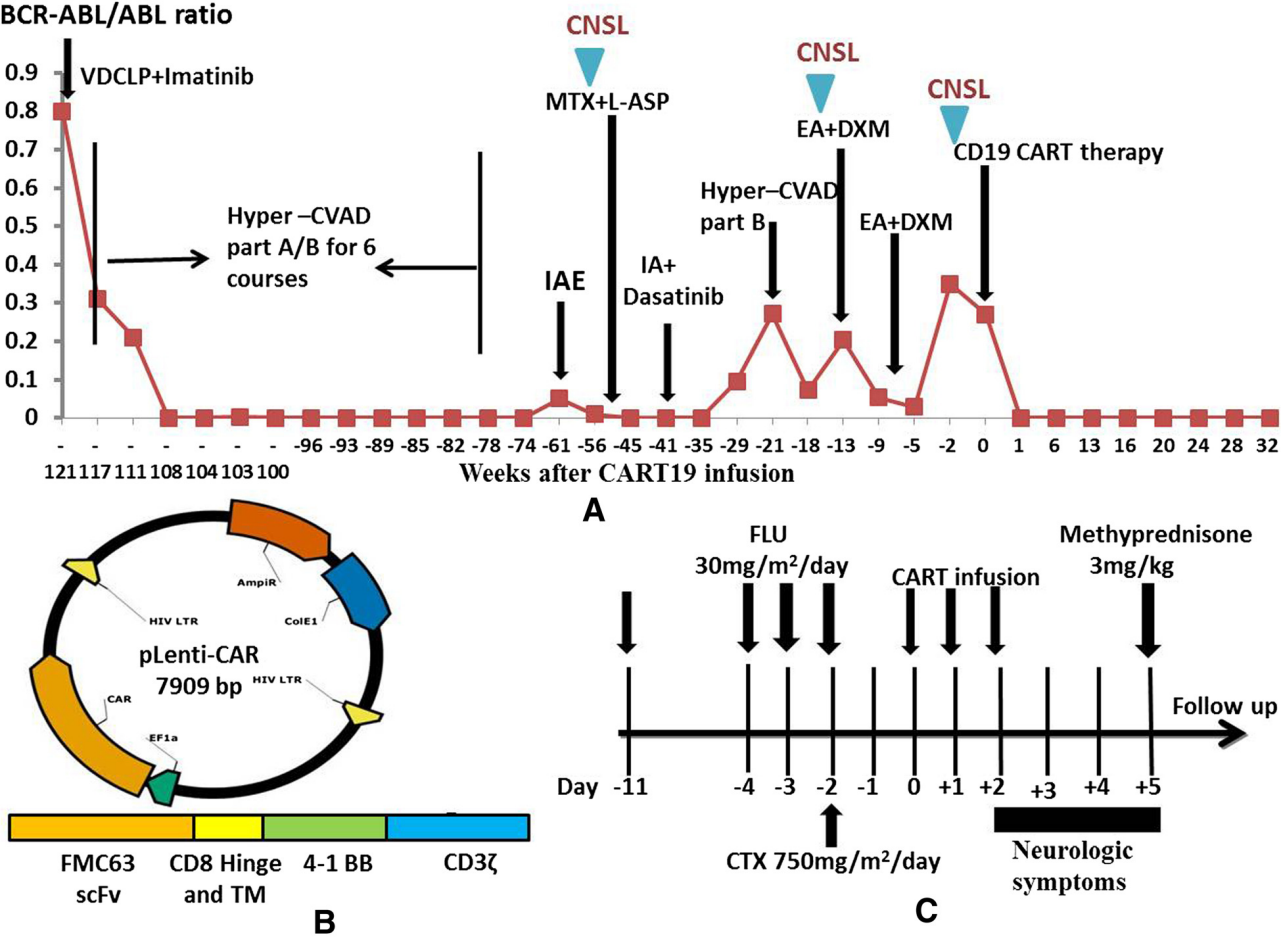
Efficacy of chemotherapy and CART19 therapy in the patient (female, 43 years old). **a** Trend in BCR-ABL/ABL ratio after chemotherapy combined with tyrosine kinase inhibitor (TKI) treatment and CART therapy. The patient underwent courses of VDCLP (vincristine, daunomycin, cyclophosphamide, asparaginase, and dexamethasone), hyper-CVAD part A (cyclophosphamide, vincristine, doxorubicin, and dexamethasone), hyper-CVAD part B (methotrexate and cytosine arabinoside), IAE (idarubicin, cytosine arabinoside, and etoposide), MTX (methotrexate) + l-ASP (l-asparaginase), IA (idarubicin and cytosine arabinoside), and EA (etoposide and cytosine arabinoside) + DXM (dexamethasone) chemotherapy. Three times of the central nervous system lymphoma (CNSL) were also indicated. About 15 times of intrathecal chemotherapy with methotrexate, cytosine arabinoside, and DXM without cranial irradiation were performed before CART19 infusion. Her cerebral spinal fluid (CSF) contained no white blood cells (WBCs) and normal level of protein when she was recruited for the CART19 clinical trial. Before CART19 infusion, 10.7 % cells remaining in the marrow were CD19+ leukemia cells, and BCR-ABL/ABL ratio in the marrow was 27 %. **b** Lentiviral vector applied to transfect T lymphocytes from the patient. A pseudotyped clinical-grade lentiviral vector including anti-CD19 scFv derived from FMC63 murine monoclonal antibody, human CD8α hinge, and transmembrane domain and human 4-1BB and CD3ζ-signaling domains was constructed. **c** Procedure of CART19 manufacture and the clinical application scheme. Lymphocyte-depleting chemotherapy regimen FC consisted of FLU (fludarabine) 30 mg/m^2^ days 1 to 3 and CTX (cyclophosphamide) 750 mg/m^2^ day 3

**Fig. 2:**
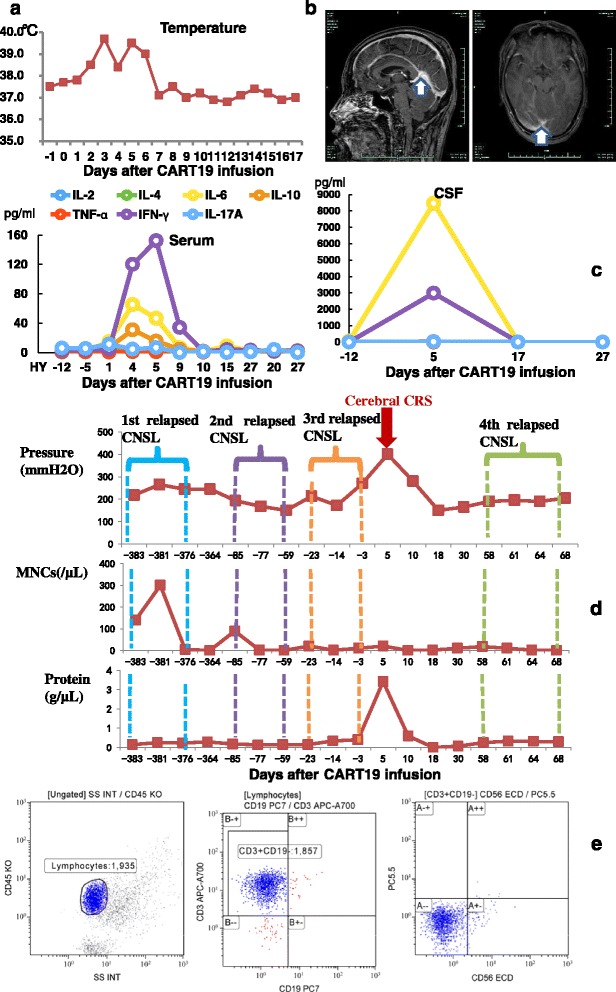
Predominant cerebral cytokine release syndrome (CRS) manifestations after CART19 therapy. **a** Wave changes in body temperature after CART19 infusion, with a maximum temperature per 24-h period indicated by the *squares*. **b** Heterogeneous structures with punctiform nodal or linear enhancement in the cerebellar sulcus and hidden parts (*arrows*) by contrast-enhanced MRI on day 5 after CART19 infusion, indicating the local infiltration of inflammatory cells. **c** Inflammatory cytokine levels in peripheral serum and CSF, respectively, at different time points. CSF cytokine concentrations were extremely higher than in the serum, with IFN-γ levels 20 times higher and IL-6 levels 190 times higher. **d** Mononuclear cell (MNC) counts and protein levels in CSF and profiles of cerebrospinal pressure at different times of CNSL and cerebral CRS after CART19 infusion. As indicated, the patient suffered three times of recurrent CNSL, with the highest MNC counts (300/μL) and protein levels (0.19 g/μL) in CSF and a higher cerebrospinal pressure (265 mmH^2^O). During CRS, the CSF contained higher levels of protein (4.0 g/μL) and less amount of WBCs (20/μL). **e** CSF cells were predominantly CD3+ T cells with few CD19+ B cells by FACS analysis which did not support a diagnosis of CNSL. For leukoencephalopathy, MRI imaging usually discloses bilateral and symmetric white matter areas of hyperintense signal on T2-weighted and fluid-attenuated inversion recovery images and signs of restricted diffusion; CSF usually contains a slight increase of WBC counts and protein levels. Thus, leukoencephalopathy was not considered. Routine CSF cultivation and specific virus DNA detection excluded bacteria, virus, and fungal infection

In the current study, the patient had experienced recurrent CNSL before CART19 infusion. Since CNS tissues lack CD19 expression [9], the CNS complications might less probably result from direct interactions between CD19+ cells and CART19. The predominance of CART19 in CSF and the huge discrepancies in inflammatory cytokine distributions strongly indicated the development of a cerebral CRS, presumably featured as CSF cytokines largely in situ produced by BBB-penetrating CAR T cells. Although no evidence of WBCs in CSF was observed before CART19 infusion, potent minimal residual CD19+ leukemia cells less than detection limits might enter CNS [10] and dramatically trigger activation of CAR T cells. Notably, this patient complicated severe cerebral CRS rather than systemic compromisations, suggesting that systemic and cerebral CRS were independent processes.

We have to acknowledge the comprehensive roles of CRS in CAR T cell therapy. Theoretically, cerebral CRS might facilitate elimination of CNSL and also cause severe CNS complications. Therefore, the timing and strategy to terminate CRS should be deliberately evaluated in clinical practice. Previous reports showed neurologic toxicities were not prevented by cytokine blockade via tocilizumab [6–8] due to its incapability of crossing the BBB. Our experience of using glucocorticoid as a salvage option could be instructive for a quick control of the severe CRS.

In all, we reported for the first time the development and control of cerebral CRS triggered by BBB-penetrating CAR T cells. This study provided insights into the etiology, diagnosis, and treatment of CRS after CAR T cell therapy.

## Abbreviations

ALL, acute lymphocytic leukemia; BBB, blood-brain barrier; BM, bone marrow; CAR T cell, chimeric antigen receptor-modified T cell; CART19, CD19-directed CAR T cell; CNSL, central nervous system leukemia; CR, complete remission; CRS, cytokine release syndrome; CSF, cerebrospinal fluid; MRD, minimal residual disease; PB, peripheral blood; WBCs, white blood cells

## Additional files

Additional file 1: Detailed patient information and procedures of diagnosis and treatment. (DOCX 17.2 KB) Additional file 2: Preparation of chimeric antigen receptor-modified (CAR) T cells targeting CD19 (CART19). (DOCX 16.7 KB)

## References

[1] Maude SL, Frey N, Shaw PA, Aplenc R, Barrett DM, Bunin NJ (2014). Chimeric antigen receptor T cells for sustained remissions in leukemia. N Engl J Med.

[2] Brudno JN, Somerville RP, Shi V, Rose JJ, Halverson DC, Fowler DH. Allogeneic T cells that express an anti-CD19 chimeric antigen receptor induce remissions of B-cell malignancies that progress after allogeneic hematopoietic stem cell transplantation without causing graft-versus-host disease. J Clin Oncol. 2016. 10.1200/JCO.2015.64.5929 PMC4872017 26811520

[3] Lee DW, Kochenderfer JN, Stetler-Stevenson M, Cui YK, Delbrook C, Feldman SA (2015). T cells expressing CD19 chimeric antigen receptors for acute lymphoblastic leukaemia in children and young adults: a phase 1 dose-escalation trial. Lancet.

[4] Han EQ, Li XL, Wang CR, Li TF, Han SY (2013). Chimeric antigen receptor-engineered T cells for cancer immunotherapy: progress and challenges. J Hematol Oncol.

[5] Maus MV, Grupp SA, Porter DL, June CH (2014). Antibody-modified T cells: CARs take the front seat for hematologic malignancies. Antibody-modified T cells: CARs take the front seat for hematologic malignancies. Blood.

[6] Kochenderfer JN, Dudley ME, Kassim SH, Somerville RP, Carpenter RO, Stetler-Stevenson M (2015). Chemotherapy-refractory diffuse large B-cell lymphoma and indolent B-cell malignancies can be effectively treated with autologous T cells expressing an anti-CD19 chimeric antigen receptor. J Clin Oncol.

[7] Turtle CJ, Hanafi LA, Berger C, Gooley TA, Cherian S, Hudecek M (2016). CD19 CAR-T cells of defined CD4+:CD8+ composition in adult B cell ALL patients. J Clin Invest.

[8] Davila ML, Riviere I, Wang X, Bartido S, Park J, Curran K (2014). Efficacy and toxicity management of 19-28z CAR T cell therapy in B cell acute lymphoblastic leukemia. Sci Transl Med.

[9] Uckun FM, Jaszcz W, Ambrus JL, Fauci AS, Gajl-Peczalska K, Song CW (1988). Detailed studies on expression and function of CD19 surface determinant by using B43 monoclonal antibody and the clinical potential of anti-CD19 immunotoxins. Blood.

[10] Del Principe MI, Maurillo L, Buccisano F, Sconocchia G, Cefalo M, De Santis G (2014). Central nervous system involvement in adult acute lymphoblastic leukemia: diagnostic tools, prophylaxis, and therapy. Mediterr J Hematol Infect Dis.

