# Supplementary material for: Predominant cerebral cytokine release syndrome in CD19-directed chimeric antigen receptor-modified T cell therapy
Source: J Hematol Oncol. 2016 Aug 15;9:70. doi: 10.1186/s13045-016-0299-5 (PMC4986179; doi:10.1186/s13045-016-0299-5)
Supplement: Additional file 1: — Detailed patient information and procedures of diagnosis and treatment. (DOCX 17.2 KB) [file 13045_2016_299_MOESM1_ESM.docx]

**Detailed general information of the patient**

A female patient aged 43 presenting with bleeding blots in the skin was diagnosed of acute lymphocytic leukemia (ALL) with BCR/ABL p210(+) in May 2013 before received a conventional chemotherapy and Imatinib Mesylate treatment. A durable remission with negative minimal residual disease (MRD) had been sustained before the first relapse 14 months after diagnosis. She had a second remission after reinduction chemotherapy and dasatinib treatment but complicated with recurrent central nervous system leukemia (CNSL) for 3 times. Therapeutic intrathecal injection with cytarabine and methotrexate for each recurrent CNSL achieved CR. Afterwards she underwent 2 times of relapse in bone marrow. She showed no response to further chemotherapy, including cytarabine, teniposide and dexamethasone after the third relapse (Fig.1A). Then the patient was recruited for CART19 clinical trial. Peripheral-blood mononuclear cells (PBMCs) were collected before administration of lymphocyte-depleting chemotherapy regimen FC (Fludarabine 30mg/m^2^ day 1 to 3, Cyclophosphamide 750mg/m^2^ day 3).
