# Supplementary material for: Predominant cerebral cytokine release syndrome in CD19-directed chimeric antigen receptor-modified T cell therapy
Source: J Hematol Oncol. 2016 Aug 15;9:70. doi: 10.1186/s13045-016-0299-5 (PMC4986179; doi:10.1186/s13045-016-0299-5)
Supplement: Additional file 2: — Preparation of chimeric antigen receptor-modified (CAR) T cells targeting CD19 (CART19). (DOCX 16.7 KB) [file 13045_2016_299_MOESM2_ESM.docx]

**CART19s transduction, culture and detection**

The single chain fragment variable (scFv) sequence specific for CD19 was derived from Clone FMC63. CAR19-4-1BBz vectors harboring anti-CD19 scFv and human 4-1BB and CD3ζsignaling domains were cloned into a lentiviral backbone. A pseudotyped, clinical-grade lentiviral vector was produced according to current good manufacturing practices. Anti-CD3 monoclonal antibody (OKT3) stimulated PBMCs were used to produce CART cells by further adding anti-CD3&CD28 antibody conjugated microbeads (Dynabeads) in culture medium containing interleukin-2 (IL-2) (500 U/mL). Lenti-virus mediated CAR transduction was performed 24 hours after cell culture and the cells were cultured and expanded for up to 10 days prior to harvest and preparation for infusion. Microbeads were removed by passing the magnetic field at the end of cultivation. Flow cytometry was used to characterize surface expression of CD19 CAR-T cells with antibodies of the anti-hCD3 FITC, anti-hCD19 APC, anti-hCD45 PE, anti-hCD4 APC, anti-hCD8 PE, anti-hCD28 APC, PE strept-avidin (BD Bioscience). The transduction efficiency was determined by FACS using goat-anti-mouse F(ab’)2 antibody (Jackson Immuno Research). Quantitative polymerase-chainreaction (PCR) and flow cytometrics analysis were performed to detect CART cells in blood and bone marrow. Soluble-factor analysis was performed with the use of serum from whole blood.

Quantification of soluble cytokine factors was performed with the use of cytometric bead-array technology and reagents from Becton Dickinson Company.

**CART19 infusion**

The patient received CART19 infusion with a total dose of 4.1×10^7^ CD3+ T cells per kilogram (6.7×10^6^ CART19 per kilogram), given over a period of 3 consecutive days.
